# Serological Evidence of Zika Virus Infections in Sudan

**DOI:** 10.3390/v16071045

**Published:** 2024-06-28

**Authors:** Awadalkareem Adam, Robert Wenzel, Elisabeth Unger, Sven Reiche, Christian Jassoy

**Affiliations:** 1 Institute for Medical Microbiology and Virology, Faculty of Medicine, University of Leipzig Medical Center, University of Leipzig, 04103 Leipzig, Germany; awadalim@yahoo.com (A.A.);; 2 Department of Experimental Animal Facilities and Biorisk Management, Friedrich-Loeffler-Institut, 17493 Greifswald-Insel Riems, Germany

**Keywords:** Zika virus, Sudan, seroepidemiology, neutralizing antibodies

## Abstract

Little is known about the frequency of Zika virus (ZIKV) infections in Sudan. The aim of this study was to obtain data on the prevalence of ZIKV infections and the immunity of the population in the country. To this end, 198 sera obtained between December 2012 and January 2013 in different regions in Sudan were examined for neutralizing antibodies against ZIKV, dengue virus (DENV), and yellow fever virus (YFV). The sera were non-randomly selected. The neutralization titers were compared with each other and with the WHO 1st International Standard for anti-Asian lineage Zika virus antibody. Twenty-six sera neutralized ZIKV. One-third of these sera had higher neutralization titers against ZIKV than against DENV-2 and -3. Two sera showed higher neutralization titers than the WHO standard for ZIKV antibodies. These data suggest occasional ZIKV infections in Sudan. The low percentage of sera in this cohort that neutralized ZIKV indicates that, in the study period, the population was susceptible to ZIKV infection.

## 1. Introduction

Zika virus (ZIKV) is a mosquito-borne virus from the flaviviridae family. This family includes arboviruses such as dengue virus (DENV), yellow fever virus (YFV), West Nile virus (WNV), tick-borne encephalitis (TEB), and Japanese encephalitis (JEV), among others [1]. Commonly, Zika virus is transmitted to humans by *Aedes aegypti* and *albopictus*; however, sexual transmission has also been reported [2]. In the past decade, the Zika virus has emerged as a clinically important virus, resulting in a pandemic in the Americas in 2015 [3]. Most human ZIKV infections are asymptomatic and self-limiting. Symptomatic infection is usually mild. However, it may inflict severe neurological manifestations such as Guillain–Barre syndrome or encephalitis. In pregnant women, ZIKV infections can result in spontaneous abortion, fetal infection, and congenital Zika syndrome, with fetal growth retardation and microcephaly [4].

There is evidence of Zika virus (ZIKV) infections in several African countries, but not in Sudan. However, it is known that Aedes mosquitoes, which transmit ZIKV, are present in the country [5]. Recently, a significant proportion of sera from multiple regions of Sudan were reported to contain ZIKV-binding antibodies. One of the sera also contained ZIKV-neutralizing antibodies [6]. Since sera from Sudan also react with other flaviviruses such as dengue virus (DENV), West Nile virus, and yellow fever virus [7,8], and antibody cross-reactions between flaviviruses are common, there is a need for further studies to better characterize the significance and extent of ZIKV infections in Sudan. Hence, the objective of this study was to identify autochthonous ZIKV infections in a sample cohort from eastern and central Sudan. In addition, we explored what proportion of the sera tested positive for neutralizing antibodies, thus suggesting effective active immunity in the subjects.

In general, the differentiation of infections with individual flaviviruses by serological tests is challenging in areas where more than one flavivirus is endemic due to cross-reactivity. To date, there is no antibody-based test algorithm that detects previous ZIKV infection beyond doubt [9]. Virus neutralization tests are considered the most specific tests for the differentiation of flavivirus infections in convalescent sera [10]. However, the diagnostic specificity of ZIKV neutralization tests in DENV-infected individuals depends on the time point after infection and the frequency of DENV infections. For instance, it was reported that after primary DENV infection, sera did not cross-neutralize ZIKV [11]. In comparison, ZIKV cross-neutralization was frequently seen in the first three months after a repeat DENV infection [12,13]. After more remote DENV infection, 18.7–23% of the sera cross-neutralized ZIKV [11,14]. The WHO considers neutralization tests useful to confirm clinical cases of infection in the laboratory; it was suggested that neutralizing antibody titers can distinguish ZIKV from DENV infections when testing for both simultaneously [12,15]. In addition, neutralization tests have been used in numerous studies on the spread of ZIKV infection [6,16,17,18,19]. Therefore, we performed neutralization tests for ZIKV as well as DENV in this study.

## 2. Materials and Methods

### 2.1. Cohort Selection and Reference Sera for ZIKV Antibodies

Serum samples were collected in December 2012 and January 2013 from patients symptomatic with fever visiting outpatient clinics in five cities in Red Sea, Kassala, and North Kordofan states, in eastern and central Sudan [7,8]. From this cohort, we purposively selected 106 Panbio DENV IgG Indirect ELISA-positive and 92 IgG ELISA-negative sera. The sera were from a previous study, but the selection of sera for this study was purposive and is not representative of the original sample.

The 1st International Standard for anti-Asian lineage Zika virus antibody (serum, NIBSC code no. 16/352) and the working reagent for anti-Zika virus antibody (plasma, NIBSC code no. 16/320) were used as reference reagents. The reference sera were obtained as lyophilized samples from the National Institute for Biological Standards and Control, Potters Bar, Hertfordshire, UK. Lyophilized samples were reconstituted with water, aliquoted, and frozen at −20 °C until usage. The International Standard contains 1000 IU/mL ZIKV neutralizing activity, and the NIBSC Working reagent contains 2756 IU/mL [20,21].

### 2.2. Dengue Virus IgG Antibody Test

Sera were examined for IgG antibodies using the Panbio Dengue IgG Indirect ELISA (Abbott Laboratories GmbH, Hannover, Germany). The test was performed in duplicates.

### 2.3. Flavivirus Neutralization Tests

ZIKV (strain BRA/2016/FC-DQ60D1-URI), DENV-1 (isolate 2522/10), DENV-2 (isolate 3229/11), DENV-3 (isolate 3140/09), and DENV-4 (isolate 3274/0) were provided by J. Schmidt-Chanasit, Bernhard Nocht Institute, Hamburg. The yellow fever virus (YFV) strain 17D-204 was cultured from a vaccine dose (Stamaril, batch no. Z5042-1, Sanofi-Aventis, Paris, France). Neutralization assays were performed as previously described [7,8]. Briefly, Vero VFM cells (25,000/well) were seeded in 96-well cell culture plates and incubated at 37 °C overnight. For neutralization screening tests, sera were diluted 1:20 in DMEM with 1% FCS. To determine the 50% neutralization titer (NT_50_), sera were twofold diluted in medium from 1:20 to 1:1280. WHO reference reagents were twofold diluted from 1:80 to 1:5120. Sera were incubated with virus for 1 h and added to the cells. Screening tests were performed in triplicates, NT_50_ determinations were performed in duplicates. The following controls were used: Vero cells without virus, virus diluted with a negative serum, and a twofold back-titration of the virus to determine the 50% tissue culture infectious dose (TCID_50_) used in the test.

After 7–8 days, cell culture medium was removed. Cells were washed twice with 200 µL phosphate-buffered saline (PBS) and fixed with ice-cold methanol for 10 min. Methanol was removed and the plates were dried in air. The plates were washed with water and the wells were blocked for 20 min with PBS containing 3% bovine serum albumin. The blocking solution was discarded and the Zika virus capsid-specific murine monoclonal antibody E4-57 (prepared in our laboratory, 1 or 2 µg/mL) or the flavivirus group antibody 4G2 (for DENV and YFV neutralization tests, 2 µg/mL, hybridoma HB-112, ATCC) were added in staining solution for 1 h. The antibody solutions were discarded, and the plates were washed 4 times with deionized water and PBS containing 0.1% Tween-20 (PBS-T). Rabbit-anti-mouse IgG/HRP antibody (diluted 1:1000, DAKO, product no. P0260) in staining solution was added to the wells and incubated for 1 h. The plates were washed 6 times with water and PBS-T. TMB substrate (TMB Soluble Reagent, TM4500, ScyTek Laboratories, Inc., Logan, UT, USA) was added for 3 to 15 min. The enzymatic reaction was stopped with 0.5 M H_2_SO_4_. All incubations were maintained at room temperature. The plates were read with a photometer at a wavelength of 450 nm and a reference wavelength of 570 nm.

The TCID_50_ of the viral input was calculated using the Spearman–Karber equation. Tests were valid when the viral titer was between 8 and 64 TCID_50_/test. The mean of the OD in control wells of Vero cells without virus plus 3 standard deviations was considered as the cut-off for infection. OD values above the cut-off indicated infection and a lack of neutralization. In the screening assay, sera that neutralized the virus in at least two of three wells were considered positive. Sera that neutralized the virus in one well were defined as borderline, and sera without exhibiting any neutralization in all three wells were negative. The NT_50_ was calculated using the Spearman–Karber equation.

### 2.4. Calibration Curve for ZIKV Neutralization Units

An antibody neutralization standard calibration curve was established with the WHO 1st International Standard. The International Standard was twofold diluted from 1000 IU/mL to 15.6 IU/mL. Each serum dilution was tested in the neutralization test and the NT_50_ was determined for each dilution. NT_50_ and IU/mL values for each serum dilution were plotted as a graph, and a linear regression curve was built.

### 2.5. Statistical Analyses

Mean titers were determined using Microsoft Excel software. The *t*-tests and Mann–Whitney U tests were performed with Social Science Statistics software (https://www.socscistatistics.com, accessed multiple times until 11 June 2024).

## 3. Results

### 3.1. Identification of ZIKV-Neutralizing Sera

**Figure 1:**
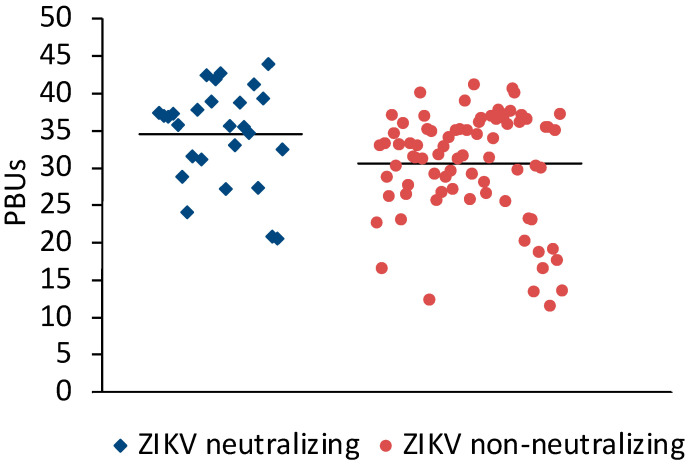
Comparison of DENV ELISA Panbio units obtained with ZIKV-neutralizing and non-neutralizing sera. Bars represent the arithmetic means. The values were significantly different (*t*-test for independent variables, *p* < 0.0001).

Sera (N = 198) from Sudan that were positive (N = 106) or negative (N = 92) in the Panbio Dengue Indirect IgG ELISA were selected for the study. A microneutralization assay for ZIKV was established to measure the neutralizing antibody response against ZIKV. Twenty-three of the DENV IgG ELISA-positive and three of the DENV IgG-negative sera neutralized ZIKV (Table 1). On average, ZIKV-neutralizing sera had slightly higher Panbio units in the DENV IgG ELISA than ZIKV non-neutralizing sera (Figure 1).

### 3.2. Comparison of the Neutralizing ZIKV Antibody Response with That of Standard Sera

The WHO 1st International Standard for anti-Asian lineage Zika virus antibody and the NIBSC Zika virus antibody working reagent were tested in the ZIKV microneutralization assay to determine the NT_50_ values that correspond to the activity of the standard reagents in IU/mL. The International Standard showed an NT_50_ of 1:422. The Working reagent had a NT_50_ of 1:1040. The reference reagents also neutralized the four dengue viruses and YFV (Table 2).

**Figure 2:**
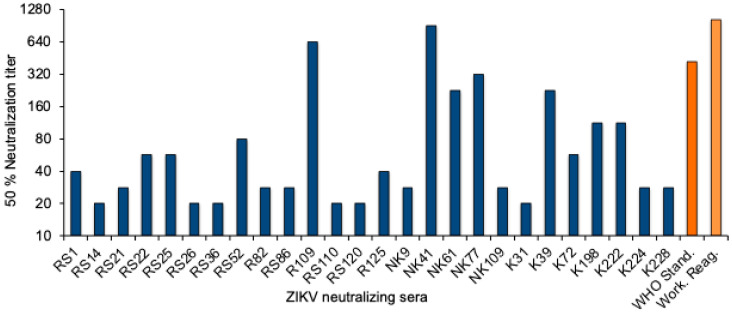
ZIKV neutralizing antibody titers. NT_50_ values of the 26 ZIKV-neutralizing sera (blue), the WHO 1st International Standard for anti-Asian lineage Zika virus antibody, and the NIBSC Working reagent for anti-Zika virus antibody (orange).

To quantitate the Zika virus neutralizing activity, a standard curve was established with the WHO 1st International Standard. The NT_50_ values were determined for twofold dilutions of the standard reagent, and linear regression analysis was performed to obtain a formula for the calculation of the Zika virus antibody activity in serum samples (Supplementary Figure S1). The equation was used to convert the NT_50_ values into international units (IU)/mL. The NT_50_ values of the 26 neutralizing sera from Sudan ranged from 20 to 905, and the activity was between 22 and 1861 International Zika virus antibody units/mL (Figure 2 and Table 3).

### 3.3. Comparison with DENV and YFV Neutralization

**Figure 3:**
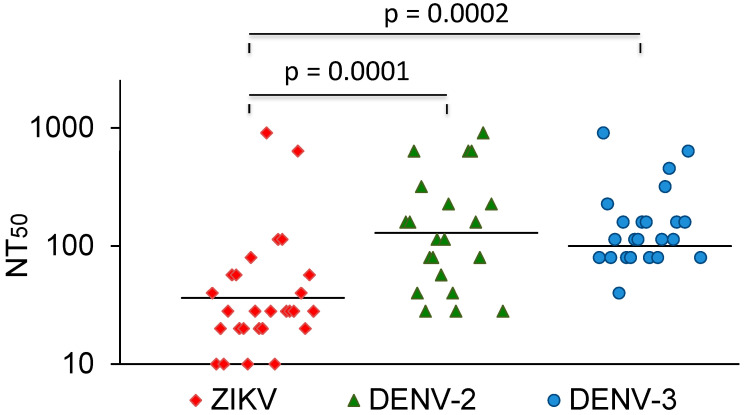
ZIKV, DENV-2, and DENV-3 NT50 values of ZIKV-neutralizing sera. Bars represent the geometric mean titres. The values were compared with the Mann–Whitney U test.

The vast majority of sera that neutralized DENV did not cross-neutralize ZIKV. In comparison, most Zika-virus-neutralizing sera also neutralized dengue viruses. A few of these also neutralized YFV. The ZIKV NT_50_ values were compared with the NT_50_ values for DENV-2 and -3. The average NT_50_ values for DENV-2 and DENV-3 were higher than the NT_50_ values for ZIKV (Figure 3). Three sera with strong ZIKV-NT_50_ values (NK41, 61, and 77) only weakly neutralized the DENVs or not at all. Two of these were also negative in the DENV IgG ELISA (Table 3).

## 4. Discussion

ZIKV was first isolated in Uganda in 1947, and was described as a new virus in 1952 [22]. Since then, transmission of the virus has been detected in several other African countries [5,23]. In Sudan, clear evidence for ZIKV infections is lacking, but there was a study in which ZIKV antibodies were found in sera from different parts of the country using an antibody ELISA. In addition, one of the sera neutralized the virus [6]. The aim of our study was to investigate this observation further. To this end, 198 sera were selected from a sample cohort and examined: 26 of the sera neutralized the ZIKV; some sera had comparatively high ZIKV neutralization titers; and 3 ZIKV-neutralizing sera contained no or minimal DENV-neutralizing or -binding antibodies. These findings are a further indication of ZIKV infections in Sudan. The data also suggest that such infections were rare.

Most sera in our study that neutralized dengue viruses did not neutralize the ZIKV. Similar observations were also made in earlier studies. ZIKV cross-neutralization was observed in fewer than 25% of the sera after remote dengue virus infections, and no cross-neutralizing antibodies were observed after a primary DENV infection [11,14]. It is not yet known how often ZIKV infections induce DENV cross-neutralizing antibodies. The observation that three of the sera tested neutralized the ZIKV but did not or hardly neutralize dengue viruses suggests that DENV cross-neutralization does not always occur.

The serum samples examined in the study were obtained in 2012 and 2013, and thus provide an insight into the situation a good 11 years ago. Since then, no outbreaks of Zika virus have been recorded in Sudan. The number and proportion of ZIKV-antibody-positive sera in the sample was rather low. Since the sera were not randomly selected, the percentage in the population cannot be extrapolated from the numbers. However, our data suggest that ZIKV-neutralizing antibodies were rare. If all subjects with detectable ZIKV-neutralizing antibodies are considered protected against disease, only a minority of the population sample would be immune. As ZIKV is transmitted by the same species of mosquito as DENV, which is endemic in many parts of Sudan, there is a risk of a future ZIKV outbreak. The three sera with strong ZIKV-NT_50_ titers without or with few DENV-neutralizing antibodies were obtained in El Obeid, in the state of North Kordofan. This suggests that this region should be given priority when looking for further evidence of ZIKV infections in Sudan.

The study has the following limitations: Firstly, the diagnostic significance of flavivirus neutralization tests for epidemiological questions is unclear. It has been reported that three quarters of the sera from persons who have had a remote DENV infection did not or only weakly neutralize ZIKV, suggesting that to some degree the neutralization assay can distinguish infections with these viruses [11,24,25]. We think that the definitive proof of ZIKV infections in Sudan requires the detection of cases according to the WHO diagnostic criteria [15]. Secondly, the sera were not randomly selected and the diagnostic sensitivity of ZIKV neutralization tests is unknown [16]. Therefore, our data lack the power and generalizability to estimate the seroprevalence of the disease and antibody-neutralizing properties on a population level.

New in the study is that the ZIKV antibody titers in sera from Sudan were compared with the 1st International Standard for anti-Asia Zika virus antibodies and converted into international units [20,21]. In the future, this will enable a comparison with ZIKV neutralization titers in other studies, provided that the WHO standard is also used as a reference.

## 5. Conclusions

These study presents serological evidence of ZIKV infections in a small number of individuals in Central Sudan. The study design did not allow calculations of the ZIKV neutralizing immunity on a population level, but the low number of ZIKV neutralizing sera suggests that during the study period the vast majority of the population was susceptible to ZIKV infections.

## Figures and Tables

**Table 1:** Number and fraction of ZIKV-neutralizing sera among selected human sera.

Selection Criteria	No. Sera	ZIKV NT Pos.
DENV IgG ELISA-positive	106	23 (21.7%)
DENV IgG ELISA-negative	92	3 (3.2%)
Total	198	26

**Table 2:** Flavivirus NT_50_ titers of the WHO 1st International Standard and the Working Reagent.

	Neutralizing Titer (NT_50_)					
Control Serum	ZIKV	DENV-1	DENV-2	DENV-3	DENV-4	YFV
1st International Standard	422	640	453	320	320	453
Working Reagent	1040	>1280	>1280	>1280	>1280	40

**Table 3:** Neutralization and NT_50_ values for ZIKV, DENV 1–4, and YFV by ZIKV-neutralizing sera from Sudan and from travellers.

	DENV IgG	ZIKV		DENV-1	DENV-2	DENV-3	DENV-4	YFV
Serum	Panbio ^1^	NT_50_	IU/mL ^2^	Screening ^3^	NT_50_^4^	NT_50_^4^	Screening ^3^	NT_50_^4^
RS1	+	40	64	+	160	80	+	<20
RS14	+	20	22	+	640	226	+	<20
RS21	+	28	39	+	320	113	+	<20
RS22	+	57	99	+	28	40	+	<20
RS25	+	57	99	n. d.	80	160	+	<20
RS26	+	20	22	n. d.	80	80	+	<20
RS36	+	20	22	+	113	80	+	<20
RS52	+	80	147	+	113	113	+	<20
RS82	+	28	39	+	n. d.	n. d.	+	<20
RSS86	+	28	39	+	226	160	+	<20
RS109	+	640	1310	+	226	160	+	<20
RS110	+	20	22	+	40	160	+	n. d.
RS120	+	20	22	+	28	80	+	<20
RS125	+	40	64	n. d.	n. d.	640	n. d.	<20
NK9	+	28	39	+	n. d.	80	+	57
NK41	+	905	1861	neg.	<20	<20	+	<20
NK61	neg.	226	450	neg.	<20	<20	equiv.	40
NK77	neg.	320	645	neg.	<20	<20	equiv.	<20
NK109	+	28	39	+	n. d.	80	+	905
K31	+	20	22	equiv.	<20	<20	neg.	<20
K39	neg.	226	450	+	160	113	+	80
K72	+	57	99	neg.	28	<20	neg.	<20
K198	+	113	215	+	640	320	+	226
K222	+	113	215	+	160	453	+	<20
K224	+	28	39	+	80	113	+	<20
K228	+	28	39	+	905	160	+	28

^1^ Panbio DENV IgG Indirect ELISA. ^2^ The IU/mL values for the serum samples except for the WHO standard and the working reagent were calculated with the formula from the standard curve. ^3^ Sera were tested at 1:20 dilution in triplicates. Pos: Neutralization in 2 or 3 of 3 wells; neg.: no neutralization; equivocal: neutralization in one of three wells. N. d.: not determined. ^4^ NT_50_: 50% neutralization titer; <20: no neutralization at 1:20 serum dilution.

## Data Availability

Dataset available on request from the authors.

## Supplementary Materials

The following supporting information can be downloaded at: https://www.mdpi.com/article/10.3390/v16071045/s1, Figure S1: Generation of a standard curve for international Zika virus antibody units. Standard serum dilutions containing 500, 250, 125, 62.5, 31, and 15.6 IU/mL were prepared from the WHO 1st International Standard for ZIKV antibodies, and the NT_50_ values were determined for each serum. NT_50_ and IU/mL values were plotted on a graph and linear regression analysis was performed. The figure shows the results of one of two experiments. Circles indicate the NT_50_ values. The dashed line shows the linear regression of IU/mL and NT_50_ values and the equation is the associated linear equation. R^2^: coefficient of determination.
