# Supplementary material for: Serological Evidence of Zika Virus Infections in Sudan
Source: Viruses. 2024 Jun 28;16(7):1045. doi: 10.3390/v16071045 (PMC11281350; doi:10.3390/v16071045)
Supplement: Supplementary file 1 [file viruses-16-01045-s001.zip › viruses-3039589-supplementary.pdf]

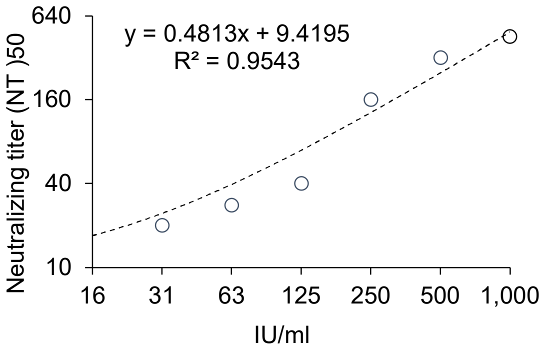


**Figure S1.** Generation of a standard curve for international Zika virus antibody units. Standard serum dilutions containing 500, 250, 125, 62.5, 31, and 15.6 IU/ml were prepared from the WHO 1st International Standard for ZIKV antibodies and the $NT_{50}$ values were determined for each serum. $NT_{50}$ and IU/ml values were plotted on a graph and linear regression analysis was performed. The figure shows the results of one of two experiments. Circles indicate the $NT_{50}$ values. The dashed line shows the linear regression of IU/ml and $NT_{50}$ values and the equation is the associated linear equation. $R^2$: Coefficient of determination.
